# In response to the Saudi healthcare reform: a cross-sectional study of awareness of and attitudes toward the public health model among health students

**DOI:** 10.3389/fpubh.2023.1264615

**Published:** 2023-10-12

**Authors:** Mohammed J. Almalki, Ali Elamin, Abdulrahman M. Jabour, Joe Varghese, Amani A. Alotaibi, Sami M. Almalki, Mohammed E. Hamdan, Maram S. Bajawi, Taym A. Alamer, Bashaier M. Alshammakhi, Hamdah H. Alabsi, Duaa H. Hassan, Hassan N Moafa

**Affiliations:** ^1^Department of Health Services Management, College of Public Health and Tropical Medicine, Jazan University, Jazan, Saudi Arabia; ^2^Department of Epidemiology, College of Public Health and Tropical Medicine, Jazan University, Jazan, Saudi Arabia; ^3^Department of Health Informatics, College of Public Health and Tropical Medicine, Jazan University, Jazan, Saudi Arabia; ^4^Department of Health Education and Promotion, College of Public Health and Tropical Medicine, Jazan University, Jazan, Saudi Arabia

**Keywords:** public health model, Saudi healthcare system, healthcare reform, health education, health students, COVID-19, Saudi Arabia

## Abstract

**Background:**

Saudi Arabia’s health sector is experiencing a significant transformation toward an emphasis on the public health model. This model is a population-based approach to preventing and controlling disease, and its importance becomes evident during infectious outbreaks and pandemics, such as COVID-19. This study aimed to assess the awareness and attitudes of health students in Jazan toward the public health model.

**Methods:**

This study applied a cross-sectional online survey. Data were collected from 3–18 November 2020 using Google Forms. A convenience sampling method was used with a final sample of 425 participants.

**Results:**

Most participants (71%) were aware of the public health model, with an average score of 11.36 out of 16. Multiple regression analysis revealed a significant association between the awareness level of the public health model and participants’ demographics, namely, gender, major of study, year of study, and prior training in public health. Participants who completed public health training (*β* = 0.220) had higher awareness scores than others. On the other hand, participants from public health (*β* = −0.342), medicine (*β* = 0.164), and nursing in Jazan (*β* = 0.128) had higher awareness of the public health model than the reference group (Nursing at Addayer College). Addayer is an area located in the rural northeast of the Jazan region. In addition, final-year students (*β* = 0.113) had higher awareness of the public health model than the reference group (year 2 pre-final students). Female participants (*β* = −0.142) had lower awareness of the public health model than male participants. Most participants (95.3%) believed that the clinical care and public health models are essential for promoting people’s health. However, 4.7% of participants believed that clinical health care is more important than public health.

**Conclusion:**

Health students, who are future healthcare professionals, must understand and value the public health model to support the planned health system reforms. It is recommended to evaluate how the education and training of students in public health, medicine, and nursing in Jazan impact the understanding and views of this cohort on the public health model compared to those of students in other health-related majors.

## 1. Introduction

The health indicators of Saudi Arabia’s population have made significant progress since the national health system was established in 1950 (1). Despite this, the prevalence of chronic diseases and related risk factors has increased substantially. According to the Saudi Arabia World Health Survey (KSAWHS) (2), health risk factors among the Saudi population include a lack of physical activity, unhealthy dietary patterns, unhealthy lifestyles, and environmental health risks. Most of the respondents (80%) undertook insufficient physical activity, 93% had an insufficient intake of fruit and vegetables, 12% were tobacco smokers, and 90% consumed two or fewer sugary drinks per day. In addition, starch food intake was higher than other food groups at 3.48 average daily servings. All households in Saudi Arabia use clean fuels for cooking, but indoor smoking occurs in 92% of households. In addition, 23% of households use incense (2). Saudi Arabia also exhibits a higher prevalence of obesity than the global average (35% vs. 13%) (3). These risk factors have led to a shift in disease patterns, posing a considerable public health concern. According to the World Health Organization (WHO), heart diseases are the major cause of death in Saudi Arabia (4). Prior research has found that non-communicable diseases, such as cardiovascular disease, cancer, chronic respiratory diseases, and diabetes, are the primary causes of death in Saudi Arabia (5, 6). Saudi Arabia also faces health risks during Hajj and Umrah, when millions of global and local pilgrims gather in Makkah and Medina for religious purposes (7–9). These annual mass gatherings present many epidemiological and environmental health challenges (9–12).

Historically, the Saudi healthcare sector has prioritized the medical model of care, which focuses on treating illnesses rather than preventing them and managing resources and personnel instead of prioritizing patient well-being (13). In addition, a significant portion of the healthcare budget is allocated to hospitals and clinical services (14). On the other hand, public health activities, including primary health services, receive less financial support (15). The findings of a recent study on the burden of disease in Saudi Arabia suggested the need for further enhancement of public health and preventive policies in the country (5).

The health sector in Saudi Arabia is currently undergoing comprehensive reform through a national transformation strategy. This reform aims to introduce a new model of care and improve healthcare quality and outcomes as a national target for the Saudi Health Vision 2030 (16). The four strategic goals of the health sector transformation strategy are facilitating access to health services, improving the quality and efficiency of health services, strengthening prevention against health risks, and enhancing traffic safety (16). The new national healthcare model is based on several principles relating to various aspects of public health. First, it aims to empower people and families to take control of their health. In addition, it will provide people with the knowledge to be well-informed regarding their health. Furthermore, it will integrate the healthcare system and services from the people’s perspective to serve their needs. Finally, it will keep people healthy by focusing on the whole population through a preventive approach rather than relying on a medical model (13, 16). The new model of care covers six subsystems, namely, keeping well (preventive care), maternity and child care, planned care, urgent care, chronic illness care, and palliative (end-of-life) care.

In support of these transformation efforts, Saudi Arabia has established a National Public Health Authority to emphasize the role of public health in the health sector transformation program. This authority aims to lead and coordinate efforts to protect and promote public health policies, behaviors, practices, and projects (17). In addition, several Saudi universities have established public health colleges to train the next generation of public health professionals. Two examples of such colleges are the King Saud bin Abdulaziz University for Health Sciences and the College of Public Health and Tropical Medicine of Jazan University (18, 19).

Furthermore, efforts have been made to develop environmental and public health policies to meet the Saudi Vision 2030 goals (13, 20, 21). For example, Saudi Arabia has adopted the “Health-in-All Policies” (HiAP) strategy. According to the WHO (22), the HiAP is “an approach to public policies across sectors that systematically takes into account the health implications of decisions, seeks synergies, and avoids harmful health impacts to improve population health and health equity.” The HiAP recognizes that policies outside of healthcare can impact population health and lead to inequalities. This approach means addressing policies such as education, transportation, housing, and finance to promote the health and equity of the population (23). Finally, the importance of public health to the Ministry of Health decision-makers is evident in recent changes, such as appointing a Deputy Minister for Public Health and renaming the “Directorate of Primary Healthcare Centers” the “Directorate of Public Health” across all health regions (24).

Despite the importance of all the efforts made by the Saudi government to improve healthcare and public health services, it will take health professionals and related parties a significant amount of time to transform the healthcare sector (25). The clinical perspectives of health clinicians often impede their comprehension of public health-wide concepts (26). Understanding public health and the differences between healthcare models is crucial for promoting well-being and illness prevention in communities as well as supporting the healthcare sector’s transformation efforts.

The medical and public health models are different healthcare approaches (27–30). The medical model focuses on the individual patient, while the public health model focuses on the entire population. The medical model is curative, aiming to diagnose, treat, and manage diseases, while the public health model is preventive, aiming to reduce disease risks and promote population health (27–30). The medical model is often seen as the dominant approach to healthcare. However, the public health model is becoming increasingly important as we face the challenges of chronic and infectious diseases and population health. The importance of the public health model was demonstrated during the COVID-19 pandemic, as awareness activities, preventive measures, and vaccinations contributed to controlling the disease in Saudi Arabia (31–36).

As the focus of our study, the public health model of care is based on public health knowledge, practices, and activities. Public health professionals lead public health initiatives to ensure and promote society’s well-being by advocating healthy behaviors, enhancing environmental health, preventing chronic and communicable diseases and injuries, addressing health threats, conducting preventive research, and introducing and implementing public health policies (37, 38). In other words, the public health model protects society from diseases by identifying risk factors and applying preventive measures (39). This model aims to build and improve health policies, conduct appropriate interventions to control risk factors, and respond immediately to health threats to minimize their long-term impact on society (40). The public health model integrates concepts from multiple disciplines, such as health education, epidemiology, health management, social care, and environmental health (39).

Despite the importance of public health as a model of care, a review of the current literature revealed a research gap in studying health students’ and health professionals’ awareness of and attitudes toward public health and its importance to the population’s health. Most published works discuss how health professionals practice public health concepts or assess their attitudes toward public health practices in specific areas. Similarly, the majority of studies regarding health students concentrate on their experiences with the public health courses they have taken. A summary of the reviewed studies is provided in the following paragraphs.

Regarding health professionals, a cross-sectional study on the importance of public health nurses in Japan found that this group has a significantly high awareness of the importance of public health activities to enhance the population’s health (39). Another recent study assessed the attitudes of Jordanian primary care nurses toward health promotion (41). Results revealed that participants had a positive attitude toward health promotion, with a mean score of 25.26 out of 32 (standard deviation = 2.96). Most participants (87.6%) agreed that nurses should play a more significant role in health promotion. However, 60.7% believed that health promotion is boring for patients. In addition, only 43.4% of respondents stated they had sufficient time to conduct health promotion activities, even though 71% believed they had the necessary abilities. Moreover, nurses with a bachelor’s degree scored higher in attitude than those with an associate degree. Likewise, a cross-sectional study of 120 Iraqi nurses showed positive attitudes toward public health activities (42). Most participants (88.3%) believed they should be more responsible for promoting health, 59.1% had sufficient time to carry out health promotion activities, and 53.3% felt confident in their abilities. In contrast, a Jordanian study (43) found that only 8% of the primary healthcare practitioners inquired about their patients’ smoking, exercise, and diet status and gave related health advice. Similarly, only 14.2% of physicians and 40.4% of non-physicians correctly identified lifestyle-related risk factors for breast, colorectal, and lung cancer. Furthermore, participants believed that patients were sufficiently knowledgeable and did not require education regarding the association between cancer and smoking (61.7%), diet (41%), or physical activity (38%). Practitioners also believed their health education would not lead to their patients quitting smoking (45.5%), taking exercise (37%), or improving their dietary habits (30%). Furthermore, 61.6% of practitioners considered counseling on preventing other non-communicable diseases more significant than counseling on cancer prevention. Finally, 40.5% of respondents agreed that lifestyle counseling would Irritate patients, and 36.5% agreed that counseling made them uncomfortable.

In terms of health students’ research, few studies have investigated attitudes toward public health activities. A cross-sectional survey assessed personal interest and attitudes toward public health activities among final-year student pharmacists in Namibia, Zambia, and Zimbabwe (44). The majority (95%) of participants expressed interest in and thought they had a responsibility for contributing to public health activities related to health promotion and disease prevention. Most participants were willing to learn more about public health activities (93% for health promotion and 89% for disease prevention). However, a minority of students believed pharmacists were utilized for public health activities in their countries. In contrast, only 9% of final-year medical students at a Sri Lankan university expressed interest in public health as a career, although 82% of participants recognized its importance. Further analysis revealed a strong correlation between career interest in public health and positive perceptions of the field, favorable experiences in the community, and a sense of competence in related skills. However, the majority of students reported feeling unmotivated to further engage with the subject matter through additional reading or review (45).

Concerning the public health curriculum, a qualitative study was conducted in Brazil to investigate the importance of public health in the education of medical students (46). Results revealed that students regarded public health as essential to their medical education and professional roles. Furthermore, those who emphasized public health more in their studies reported higher levels of satisfaction and proficiency in various aspects of public health-related work. The study suggested incorporating public health more deeply, starting early, and prioritizing practical activities to stimulate interest and attract more students (46). Similarly, a mixed-methods study examined the knowledge, attitudes, and perceptions of fifth-year medical students at South African University regarding their public health course to inform the curriculum. Most students expressed positive attitudes toward public health as a discipline, advocating for its integration into medical curricula. However, the course received negative feedback from the majority of students as they believed it lacked sufficient opportunities for learning and research and failed to incorporate practical or service-based components (47). These perceptions were supported by findings from a cross-sectional study that evaluated attitudes toward public health education among 669 medical students in the United States. A significant majority (78%) of participants strongly believed that physicians must acquire knowledge and skills related to public health (48). Moreover, beliefs about public health were similar among health students and medical students. A qualitative study was conducted to understand the perceptions of final-year allied health university students in the United Kingdom of their role in delivering public health advice. Despite their desire to provide public health advice, participants were hindered by limited knowledge and time. They proposed incorporating more public health education into the curriculum and clinical placements to overcome these obstacles (49).

At the regional level, a study was conducted in Qatar to examine how pharmacy students perceive the roles and responsibilities of pharmacists in providing public health services. The results showed that participants have positive attitudes toward pharmacists’ public health roles and responsibilities (50).

In Saudi Arabia, few studies have investigated this issue. Alshurayhi (51), in a cross-sectional study, assessed the public health knowledge of 320 medical and allied health sciences students in Riyadh. The findings indicated that 86.6% of participants think it is essential to learn about public health, and 88.4% believe it should be taught in universities. In addition, 64% believe public health directly impacts patient care and outcomes. Another cross-sectional survey, from Qassim, Saudi Arabia, examined the attitudes and knowledge of 100 pharmacy students regarding national public health programs (52). The results showed that 77% of the participants believed that pharmacists must learn about national public health programs, and 71% stated that their academic curriculum did not cover the national health policy. While only 16% of participants reported feeling adequately informed about national public health programs, a significant majority (81%) expressed a strong desire to contribute to these initiatives. Most recently, a cross-sectional study was conducted at a Saudi university in Riyadh to analyze pharmacy and nursing undergraduates’ perceptions and awareness of the public health course. Most participants (90.1%) agreed that public health courses should be part of the health college curriculum to prepare students for future careers. The study major was significantly associated with interest in public health (*p* = 0.011). Although undergraduate pharmacy and nursing students were interested in pursuing public health careers, they did not receive adequate formal education in public health (53). These studies targeted pharmacy and nursing students in the central region of Riyadh and Qassim Further research is needed on students studying various areas of health and medicine in different Saudi regions.

Understanding health students’ awareness of and attitudes toward the public health model is crucial for designing and improving academic programs and preparing future healthcare professionals. To achieve the goals and principles of the new health model in Saudi Arabia, health professionals need to have a high level of awareness as well as a positive attitude toward the role and importance of public health. Thus, this study aims to assess the awareness and attitude of Jazan University health students toward the public health model. The findings of this study will assist in identifying areas for improving public health curricula at Jazan University and preparing our graduates to contribute significantly to the new national healthcare model, which is primarily based on public health (13, 16). In addition, the findings are expected to bridge the current research gap related to university health students’ awareness of and attitudes toward the public health model in Saudi Arabia.

## 2. Materials and methods

### 2.1. Study design, population, and sample

This study used a cross-sectional design and a convenience sampling method. All health students at Jazan University were eligible to participate in this study. The study sample included male and female students enrolled in health majors at Jazan University from their first year to the internship on either urban or rural campuses. Students enrolled in majors other than health, those who discontinued or withdrew from their studies, and those who enrolled in institutions other than Jazan University were excluded from the study sample.

The sample was determined through Epi Info version 7.2.4 (54) to be 370 participants based on the total population (9500), a 50% response distribution, a 5% margin of error, and a 95% confidence interval. This study complies with Strengthening the Reporting of Observational Studies in Epidemiology (Supplementary materials).

### 2.2. Study setting

The study was conducted among health students at Jazan University, a relatively young university in the Jazan region of southern Saudi Arabia. The main campus in Jazan City comprises 16 colleges, including six health colleges, namely, Medicine, Dentistry, Pharmacy, Applied Medical Sciences, Nursing, and Public Health. The university also has several small campuses and colleges distributed across the region, including the University College in Addayer Governorate, which is located in a rural area (55). This college provides several academic majors, including nursing.

### 2.3. Questionnaire

The questionnaire for this study consisted of four parts: demographics, awareness of the public health model, attitudes toward the public health model, and an open-ended space. Demographic variables were gender, age, year of study, major of study, and whether the participant had completed public health training. The personal awareness section consisted of 16 questions focusing on the definition of public health, the general role of public health, the focus of public health professionals, the differentiation of public health from clinical care, the essential functions of public health, and the role of public health in the new national healthcare model of Saudi Arabia. These questions were mainly developed based on reliable information from a variety of scientific sources, including the Ministry of Health in Saudi Arabia, the American Public Health Association, the Centers for Disease Control and Prevention (CDC), the CDC Foundation, and the WHO Eastern Mediterranean Regional Office (16, 37, 38, 56–59).

Awareness questions were answered with “true,” “false,” or “I do not know.” In addition, the personal attitudes section consisted of three questions with two answer options for each. The first question asked whether participants believed in integrating clinical healthcare and public health models to promote population health in Saudi Arabia or if they thought the clinical healthcare model was sufficient. The second question asked whether participants believed that public health measures played a significant role in preventing infectious and chronic diseases or if they believed that these measures had little impact on these matters. The final question investigated participants’ attitudes regarding the role of public health during COVID-19, that is, whether they believed it played a significant or minimal role.

The questionnaire was initially developed in English by the first author of this study. Then, it was reviewed and approved by an English editor and two public health researchers. They ensured that the developed questions were clear and appropriate to the local content. Moreover, the first author developed an Arabic version of the questionnaire, and two bilingual public health researchers confirmed its clarity. Finally, the questionnaire was distributed to 33 individuals to ascertain the clarity and applicability of the study tool. The Arabic version was used to collect the data from participants as they were from an Arabic-speaking background. The English version was used to write and publish the findings. Additional information regarding the questionnaire versions and sections is provided in the Supplementary materials.

### 2.4. Data collection

Due to the COVID-19 pandemic, social distancing, and the shift to distance learning, we conducted an online survey *via* Google Forms from 3–18 November 2020. The survey link and information were sent to eligible students *via* email and WhatsApp groups. The public virtual studies may be biased by the selection of younger and more technologically-savvy participants, excluding older individuals and those without technology and Internet access from the sample. In our study, health students at Jazan University constitute the study population. They are all expected to be young and well-equipped with technology, as their blended learning requires the Internet and smart devices. To avoid potential sampling bias, we ensured an equal chance of inclusion by distributing the survey link to all health students at Jazan University and sending two reminder emails.

### 2.5. Ethical considerations

To maintain ethical standards for this study, we ensured that our virtual targeted participants were fully informed about the voluntary nature of their participation in the study, the voluntary consent provision, and the steps taken to protect their privacy. They received clear information on how and why to participate in the study. We assured them that their data would be kept confidential and used only for scientific research. At the beginning of the questionnaire, there was a yes/no question to confirm the student’s affiliation with Jazan University. Then, we obtained virtually informed consent from all participants using another yes/no question. The “yes” option automatically transferred the participants to the questionnaire, whereas the “no” option ended the survey form. The Jazan University Committee of Research Ethics approved this study (REC42/1/145).

### 2.6. Data analysis

IBM SPSS version 27 (60) was used to clean and analyze the collected data. We calculated and reported the descriptive statistics for demographics and other variables. Multiple regression analysis was employed to assess the association of awareness scores with the demographic variables. The awareness options were coded as follows: each correct option scored one point, and the incorrect or “I do not know” option scored zero. This coding gave a score range of 0–16, with a higher score indicating a higher level of awareness. Incomplete responses were omitted.

The demographic variables used for the statistical analyses were gender, age, year of study, major of study, and prior training in public health. These variables were coded as follows, gender (Male = 0, Female = 1), age (≤ 22 years = 0, > 22 years = 1), prior training in public health (No = 0, Yes = 1), year of study (Year 1 students = 1, Year 2–pre-final students = 2, Final-year students = 3, Internship students = 4) and major of study (medicine = 1, public health = 2, nursing in Jazan = 3, nursing in Addayer = 4, applied medical sciences = 5, and other health majors = 6). A *value of p* <0.05 for all tests was considered statistically significant.

## 3. Results

### 3.1. Demographic description

A total of 450 students submitted the survey. After excluding 25 incomplete responses, we obtained a final sample of 425 participants. The mean age for participating students was 22.03 years (SD = 1.85, range 18–30). Over half of the sample (64.5%) were 22 or younger, 79.8% were female, and 31.1% were from the Public Health College. Most participants were either in their second to pre-final academic years (48.2%) or in their final academic year (34.4%). In addition, 64.2% of the participants stated they had not attended a public health model training (Table 1).

**Table 1:** Demographic characteristics of the participants.

Variables (*N* = 425)		*n*	%
Gender	Male	86	20.2
	Female	339	79.8
Age	≤ 22 years	274	64.5
	> 22 years	151	35.5
Major of study	Medicine	55	12.9
	Public Health	132	31.1
	Nursing in Jazan	32	7.5
	Nursing in Addayer	108	25.4
	Applied Medical Sciences	49	11.5
	Other Health Majors *	49	11.5
Year of study	Year 1 student	30	7.1
	Year 2 to pre-final student	205	48.2
	Final-year student	146	34.4
	Internship student	44	10.4
Prior training in public health	No	273	64.2
	Yes	152	35.8

* The “Other Health Majors” category includes dentistry and pharmacy.

### 3.2. Awareness of the public health model

The mean awareness score toward the public health model among health students in Jazan was 11.36 out of 16 (SD = 1.78, range 7–16), indicating an overall awareness level of 71%. Students demonstrated high awareness of the public health model—over 90% in four items and over 80% in nine of 16 items. The highest-scoring questions were about health promotion and disease prevention as a public health function, followed by the definition of public health. However, four of the 16 items were answered correctly by fewer than 50% of the students, with the lowest score being 8.5% for the question about the role of clinical professionals (Table 2). Cross-tabulating these results with the year of study indicated that most of these responses were provided by junior students.

**Table 2:** Questionnaire items of awareness toward the public health model.

	Items		*N*= 425	
			Correct answers	(%)
AW.1	Definition of public health (T*).		415	(97.6)
AW.2	Public health is the science and practice of diagnosing, treating, and preventing disease at the individual level (F**,***rev.***^).		212	(49.9)
AW.3	Role of clinical professionals (F,***rev.***).		36	(8.5)
AW.4	Role of public health professionals (F,***rev.***).		248	(58.4)
AW.5	Public health works to limit health disparities by promoting healthcare equity, quality, and accessibility (T).		377	(88.7)
AW.6	Public health functions may include:			
	AW.6_1)	Surveillance and monitoring of population health (T).	389	(91.5)
	AW.6_2)	Health promotion and disease prevention (T).	418	(98.4)
	AW.6_3)	Health protection for the population, including management of environmental, food, and occupational safety (T).	380	(89.4)
	AW.6_4)	Preparedness and response to disease outbreaks, natural disasters, and other emergencies (T).	328	(77.2)
	AW.6_5)	Assuring effective health governance, public health legislation, financing, and institutional support (T).	315	(74.1)
	AW.6_6)	Training for specialists in curative medical aspects (F,***rev.***).	79	(18.6)
	AW.6_7)	Assuring a sufficient and competent workforce for effective public health delivery (T).	374	(88.0)
	AW.6_8)	Effective communication and social mobilization for health (T).	401	(94.4)
	AW.6_9)	Advancing public health research to inform and influence policy and practice (T).	375	(88.2)
	AW.6_10)	Providing patients with acute treatment measures (F,***rev.***).	128	(30.1)
AW.7	The new national healthcare model in Saudi Arabia primarily emphasizes public health aspects and practices (T).		351	(82.6)

*N* = the total number of respondents; answer options = true, false, do not know; true answer score = 1, do not know = 0, and false = 0. AW2-AW4, AW6_6, and AW6_10 are reversed-scored items. n = the number of participants who chose correct answers; % = the percentage of the “correct answers” compared to the total sample. *T = true, **F = false, ^ rev. = reversed.

### 3.3. Factors associated with public health model awareness

Multiple regression was performed to predict awareness of the public health model based on gender, age, major of study, year of study, and prior training in public health. A significant regression equation was found (*F*(11, 413) = 11.632, *p* < 0.001, *R^2^* = 0.216). Gender, major of study, year of study, and prior training in public health were significant predictors of public health model awareness. Participants who completed public health training (β = 0.220), participants from public health (β = −0.342), medicine (β = 0.164), and nursing in Jazan (β = 0.128) and final-year students (β = 0.113) had higher awareness scores regarding the public health model than the reference groups. On the other hand, female participants (*β* = −0.142) had lower awareness of the public health model than male participants (Table 3).

**Table 3:** Standard multiple regression of the demographic predictors on the awareness scores.

							95.0% CI for B	
		B	Std. Error	Beta (*β*)	t	Sig.	Lower	Upper
1 (Constant)		10.651	0.328		32.442	<0.001	10.006	11.297
Gender	1 = Female	−0.627	0.226	−0.142	−2.774	0.006	−1.071	−0.183
Age	0 = ≤ 22 yrs.	0.164	0.196	0.044	0.838	0.402	−0.221	0.550
Major of Study	1 = Medicine	0.867	0.308	0.164	2.813	0.005	0.261	1.474
	2 = Public Health	1.314	0.220	0.342	5.964	<0.001	0.881	1.747
	3 = Nursing in Jazan	0.862	0.318	0.128	2.712	0.007	0.237	1.487
	5 = Applied Medical Sciences	0.317	0.284	0.057	1.115	0.265	−0.242	0.876
	6 = Other	0.251	0.276	0.045	0.911	0.363	−0.291	0.794
Year of Study	1 = Year 1	−0.533	0.314	−0.077	−1.696	0.091	−1.150	0.085
	3 = Final-year	0.424	0.194	0.113	2.179	0.030	0.041	0.806
	4 = Internship	0.465	0.310	0.080	1.499	0.135	−0.145	1.075
Prior training in public health	1 = Yes	0.814	0.174	0.220	4.667	<0.001	0.471	1.157

Method: Enter. Dependent Variable: Total Awareness Scores. *R*^2^ = 0.237 (Adjusted *R*^2^ = 0.216). References groups were excluded automatically by SPSS: gender (0 = Male), age (1 = > 22 yrs.), major of study (4 = Nursing in Addayer), year of study (2 = Year 2 to pre-final), and prior training in public health (0 = No).

### 3.4. Attitudes toward the public health model

#### 3.4.1. Positive attitudes

**Figure 1:**
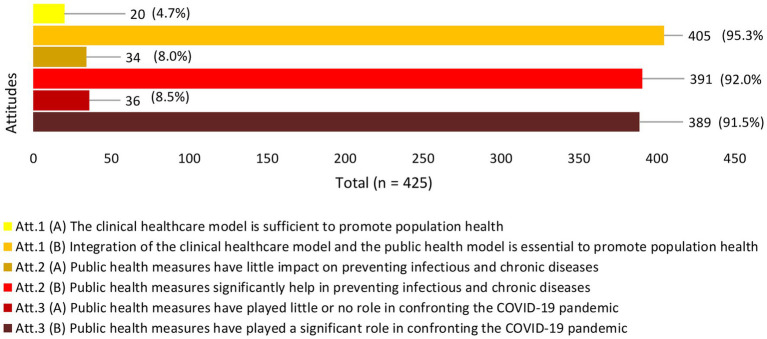
Attitudes toward the public health model.

**Figure 2:**
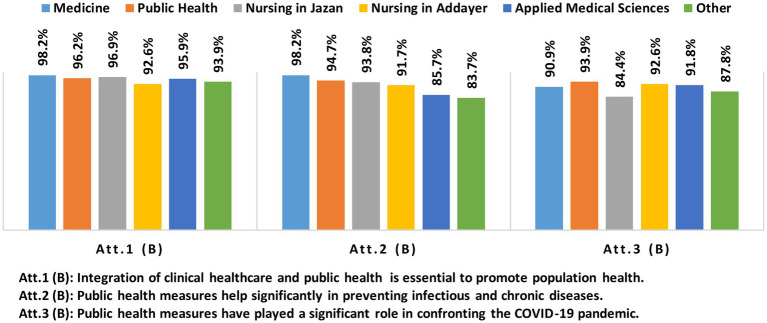
Percentages of participants with positive attitudes toward the public health model by majors.

Most participants (95.3%) agreed that both clinical care and public health models are necessary for promoting people’s health, with the highest positive attitudes observed among medical (98.2%), nursing in Jazan (96.9%), and public health majors (96.2%). Almost 92% of participants believed that public health measures significantly impact the prevention of infectious and chronic diseases. Participants from medicine, nursing in Jazan, and public health showed the highest positive attitudes, with 98.2, 94.7, and 93.8%, respectively. In addition, 91.5% of participants believed public health played a significant role in confronting COVID-19, with the highest positive attitudes among participants from public health (93.9%). Figures 1, 2 show the attitudes described in more detail.

#### 3.4.2. Negative attitudes

**Figure 3:**
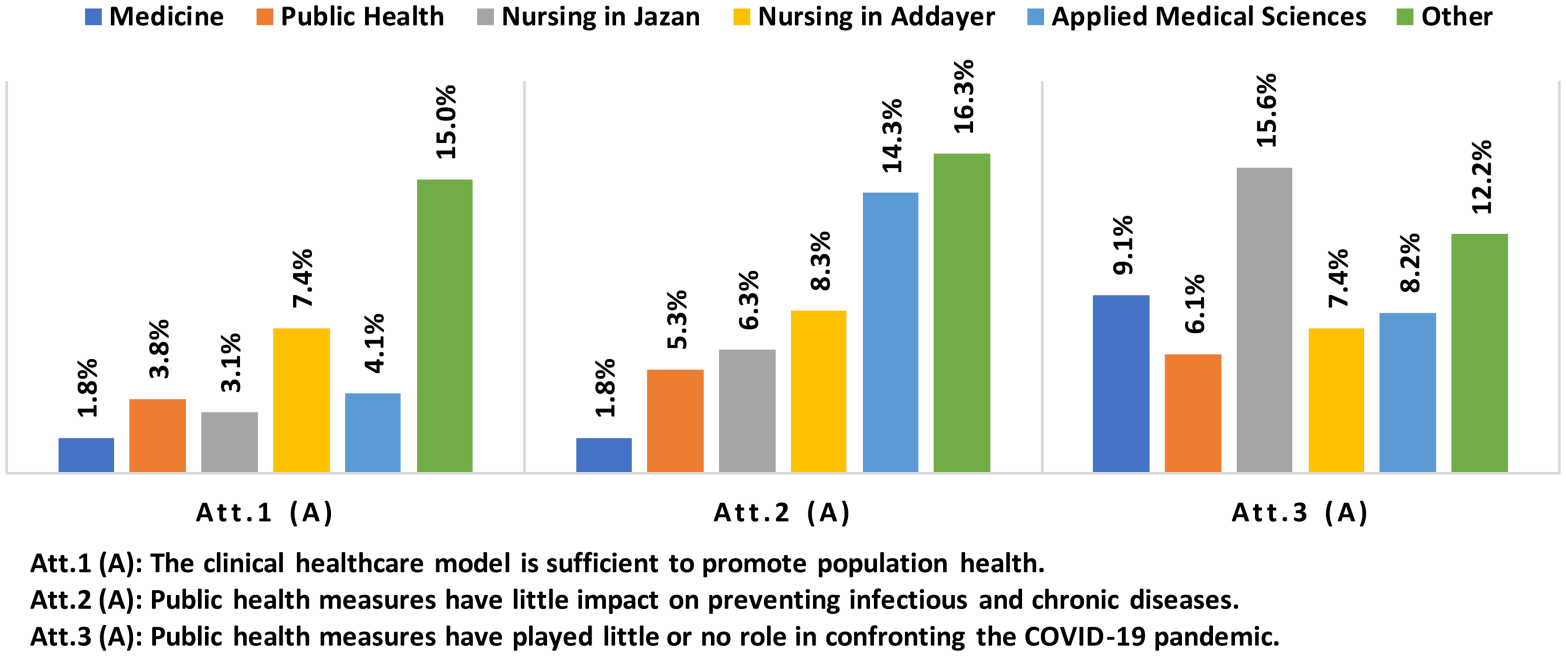
Percentages of participants with negative attitudes toward the public health model by majors.

While most participants had positive attitudes toward the public health model, a small percentage (4.7%) displayed more negative attitudes toward public health than toward clinical healthcare. Specifically, nursing students in Addayer (7.4%), the ‘other health majors’ category (6.1%), and medical sciences majors (4.1%) exhibited the highest negative attitudes toward public health. Additionally, 8% of the participants expressed a negative attitude toward the role of public health in preventing infectious and chronic diseases. This negative attitude was observed among the ‘other health majors’ category (16.3%), medical sciences (14.3%), and nursing in Addayer (8.3%). Furthermore, 8.5% of the participants reported that public health had no role in fighting COVID-19. This negative attitude was observed among participants of nursing in Jazan (15.6%), the ‘other health majors’ category (12.2%), and medical sciences (9.1%). Refer to Figure 3 for more information.

## 4. Discussion

Public health is crucial for improving population health (61). Saudi Arabia has developed a new national healthcare model to achieve its health sector transformation strategy as well as the Saudi Health Vision 2030 (13). This new healthcare model is primarily based on public health. To successfully implement the new national healthcare model and meet the growing frequency of global health emergencies and pandemics, today’s health students need to be trained in the public health model and be adequately prepared for their careers. Therefore, this study assessed Jazan University health students’ awareness of and attitudes toward the public health model.

The study results found that most participants had a high level of awareness of and positive attitudes toward the importance of public health as a healthcare model. However, a substantial portion of the participants demonstrated low levels of public health knowledge in some key areas. They answered several questions incorrectly. For example, half of the participants believe that public health is the science and practice of diagnosing, treating, and preventing disease at the individual level. Most participants had difficulty distinguishing the roles of health clinicians and public health professionals. The majority of the participants selected training for specialists in curative medical aspects as one of the essential public health functions. Nearly two-thirds of the participants reported providing patients with acute treatment measures as one of the public health essential functions. Approximately 18% of the participants lacked awareness of the role of the public health model in the new national healthcare model in Saudi Arabia. It was observed that junior respondents provided most of these responses. Although they knew the theoretical definition of public health, they were confused about its practical application. These findings suggest the need to improve public health curricula and training plans for health students, that is, future health professionals. They must be equipped with the necessary knowledge and skills as early as possible.

A significant association was found between awareness scores and participants’ demographics, including gender, major of study, year of study, and prior training in public health. The nursing students in Addayer had lower awareness than participants from public health, medicine, and nursing in Jazan. However, no significant difference was found between nursing students in Addayer and participants from applied medical sciences, dentistry, and pharmacy. Compared to other majors, public health students were expected to have a higher awareness of the public health model considering their study field. We also expected nursing students in Addayer to have a higher awareness than non-public health students because they study and train in rural areas, where many public health activities and programs are implemented. Unfortunately, this prediction was inaccurate. This finding is inconsistent with prior research (62–64) that found that providing training to internship students in rural areas helps them develop public health knowledge, skills, and experience. According to Bailey and Pit (64), medical trainees in rural places had higher levels of responsibility than urban trainees as well as more opportunities for practicing because they often work in small groups. On the other hand, students of other health majors are only based in the Jazan City campus and trained in the same urban health facilities. We anticipated that this pressure on urban health facilities would limit students’ exposure to adequate public health training.

In addition to the public health major, medicine and nursing in Jazan seem to place a greater emphasis on public health in their curricula and activities than other health majors. According to Milaat et al. (65), Jazan College of Medicine is implementing a curriculum that focuses on community-based training. However, it seems necessary to enhance public health concepts in the curricula of nursing in Addayer, applied medical sciences, dentistry, and pharmacy.

Although public health students were more aware of the public health model than other participants, a small percentage had negative attitudes toward public health. There is no apparent reason for this. A possible explanation is that health students are enrolled in majors based on their grade point average (GPA) in the first academic year. Therefore, students with higher GPAs are enrolled in medicine, dentistry, pharmacy, and nursing, while the remaining are registered in other health majors according to their GPAs. This practice may make dissatisfied students less committed to their majors. There is a significant relationship between students’ satisfaction with the study major and their aspiration levels and motivation for achievement. Satisfied students have higher aspirations, achieve better goals, and develop self-confidence and self-esteem (66–68). According to Mahmoud, Al-Zalabani and Bin Abdulrahman (24), public health programs in Saudi Arabia struggle to attract students due to a limited perception of the specialty, making public health unattractive as a career choice. Another possibility for this negative attitude is that the old study plans of the College of Public Health did not succeed in implanting the importance of public health compared to the medical model among college students. However, these interpretations remain only probabilities, requiring further investigation to understand the influencing factors.

Small portions of non-public health participants also showed negative attitudes toward public health. Negative attitudes from other non-public health students toward public health concepts could be attributed to their curricula. A recent Saudi study demonstrated a strong correlation between a student’s major and their interest in public health. However, even those interested in public health lacked sufficient formal education and training (53). Furthermore, the education and training of nursing students in Addayer focus primarily on rural communities and public health, which perhaps makes them impressed by the role of large hospitals in providing advanced clinical services. A further potential explanation is that even though they study and train in rural areas, they may perceive themselves as clinicians rather than public health nurses. There has been a rivalry between clinical and public health majors, each claiming to be responsible for enhancing a country’s health (69).

Another interesting finding is that the final-academic-year students had greater awareness than other students, including interns. The junior students were expected to have less awareness of the public health model because their study program focuses on science, English language, and introductory health courses (70). However, it is challenging to interpret the higher awareness of final-academic-year students compared to that of internship students. This result can be attributed to the new study plans adopted by the health colleges at Jazan University during the past few years (71). For example, in the College of Public Health, these plans introduced new scientific courses, promoted research, integrated practical training with theory, updated content, and organized academic requirements for all courses (19). New study plans may have played a key role in strengthening the concept of public health compared to the old study plans studied by internship students. In addition, during data collection, the intern students were in the early stage of field training and lacked the experience necessary to fill in any gaps in the public health curriculum. These interpretations need more investigation for a better understanding.

The study results also found that students who completed a training activity regarding public health were more aware of its concepts and importance than other students. This result highlights the importance of training health students in the public health model.

Curricula planners, developers, and instructors at Jazan University may benefit from these findings to improve the study, training plans, and content of health courses concerning public health. Additionally, developing field training plans, updating health curricula, and offering elective academic courses and short online courses on public health can help improve public health model awareness and attitudes among health majors.

Despite the importance of this study, it has its limitations. First, the COVID-19 pandemic limited researchers’ options for data collection due to social distancing measures and remote study arrangements for students. Second, we used a non-probability sampling technique to distribute the questionnaire, and only interested students responded. The third limitation is the unequal representation of gender. The majority of respondents were female, which could influence the generalizability of the findings. Another limitation is that we used an online survey to collect the data, leaving the interpretation of questions to the participants. This approach may have induced unintentional bias and may limit the study’s generalizability.

On the other hand, our study has several strengths. It added significant value to the body of knowledge. Saudi Arabia has experienced a variety of public health challenges, including an increase in chronic diseases (2–6) and the risk of environmental and infectious diseases from mass gatherings (7–12). These changes in disease patterns and health threats have a significant impact on the population’s health and place a heavy burden on health services and financial resources. The Saudi Arabian government has implemented health system reforms to strengthen public health concepts and practices. The success of these initiatives depends on raising awareness of, understanding of, and positive attitudes toward the public health model. This study investigated awareness of and attitudes toward the public health model in response to the current healthcare reforms. The findings have the potential to inform and enhance decision-making processes and contribute to advancing health curricula regarding the public health model. This will improve collaboration among relevant parties and help prepare qualified health professionals who value and support the public health model.

## 5. Conclusion

Most participants had high awareness levels of and positive attitudes toward the public health model. Despite this, small portions of students still need to make an effort to improve their awareness of and attitudes toward the public health model. Students in the final academic year, students of public health, medicine, and nursing in Jazan, and those who completed public health training were likely to be more aware of the public health model. The findings of this research could be used to develop appropriate strategies to improve the awareness and attitudes of health students at Jazan University toward the importance of the public health model. These strategies may include reviewing the current field training plans, developing curricula, providing elective academic courses, and providing short online courses. Such enhancement efforts will help prepare health graduates at Jazan University to contribute significantly to the new national healthcare model and other public health initiatives, especially during emergencies and pandemics.

Further research is needed to compare the current study plans and curricula with the old ones. Likewise, another study could compare the curricula, teaching methods, and training activities used in the public health, medicine, and nursing majors in Jazan with those used in nursing major in Addayer College, dentistry, pharmacy, and applied medical sciences to determine the reasons for the revealed differences in awareness of the public health model. Furthermore, the negative attitudes of some public and non-public health students toward public health raise the question of why such attitudes should exist. It is crucial to study and understand this matter. It is also recommended to assess changes in the awareness and attitudes of participating students before and after introducing related curricula, selective academic courses, or short online courses. Finally, repeating this study using a random sampling technique would be beneficial.

## Data availability statement

The raw data supporting the conclusions of this article will be made available by the authors, without undue reservation.

## Ethics statement

The Jazan University Committee of Research Ethics approved this study (REC42/1/145). The studies were conducted in accordance with the local legislation and institutional requirements. The participants provided their written informed consent to participate in this study.

## Author contributions

MA: Conceptualization, Formal analysis, Methodology, Project administration, Supervision, Visualization, Writing – original draft. AE: Formal analysis, Methodology, Resources, Writing – original draft. AJ: Conceptualization, Formal analysis, Investigation, Writing – review & editing. JV: Conceptualization, Formal analysis, Investigation, Writing – review & editing. AA: Formal analysis, Resources, Validation, Writing – review & editing. SA: Data curation, Project administration, Resources, Visualization, Writing – review & editing. MH: Conceptualization, Project administration, Writing – review & editing. MB: Conceptualization, Methodology, Resources, Writing – review & editing. TA: Investigation, Methodology, Resources, Writing – review & editing. BA: Conceptualization, Methodology, Resources, Writing – review & editing. HA: Conceptualization, Data curation, Investigation, Resources, Writing – review & editing. DH: Conceptualization, Investigation, Resources, Writing – review & editing. HM: Conceptualization, Methodology, Validation, Writing – review & editing.
