# Supplementary material for: In response to the Saudi healthcare reform: a cross-sectional study of awareness of and attitudes toward the public health model among health students
Source: Front Public Health. 2023 Oct 12;11:1264615. doi: 10.3389/fpubh.2023.1264615 (PMC10602726; doi:10.3389/fpubh.2023.1264615)
Supplement: Supplementary file 1 [file Data_Sheet_1.PDF]

Note: Researchers may use and modify this questionnaire without prior permission as long as they cite the source.

Supplementary Material

Supplementary Materials: Questionnaire of Awareness and Attitudes Toward Public Health Model

Questionnaire of Awareness and Attitudes Toward Public Health Model

Dear health students at Jazan University in Saudi Arabia,

This survey aims to assess the awareness and attitudes of health students in Jazan toward the public health model in response to the recent reforms in the Saudi healthcare system. We look forward to your participation. Taking part in this study is voluntary anonymous. Data from this tool will be used for research purposes only according to the requirement of the Research Ethics Committee at Jazan University (Approval: REC42/1/145). Answering all questions will enable us to analyze this questionnaire's data and obtain accurate findings. Finally, filling out the questionnaire takes only about three minutes.

For Further Information, you may contact:
Mohammed Almalki (PI)
College of Public Health and Tropical Medicine
Jazan University
mjalmalki@jazanu.edu.sa

Thank you very much.

ARE YOU A HEALTH STUDENT AT JAZAN UNIVERSITY?

☐ No (you may stop here, thank you)
☐ Yes (please go to the next question)

I AM 18 YEARS OR OLDER, UNDERSTAND THIS RESEARCH'S AIM, AND AGREE TO PARTICIPATE VOLUNTARILY.

☐ No (you may stop here, thank you)
☐ Yes (please start the questionnaire)

PART ONE:
BACKGROUND INFORMATION

A. Gender:
☐ Male ☐ Female

B. Age: (__________________)

C. College of study: (_____________________)

D. Major of study: (_____________________)

E. Academic level (Year of study):
☐ Year 1
☐ Year 2 to pre-final
☐ Final year
☐ Internship

F. I received training in public health:
☐ No ☐ Yes

PART TWO:
PERSONAL AWARENESS REGARDING THE PUBLIC HEALTH MODEL (please answer according to your knowledge without referring to any scientific sources)

AW1: Public health can be defined as the science of protecting and enhancing the general population's health through promoting healthy lifestyles, improving environmental health, preventing chronic and infectious diseases and injury, responding to health threats, conducting preventive research, and introducing public health policies.
☐ True
☐ False
☐ I do not know

AW2: Public health is the science and practice of diagnosing, treating, and preventing disease at the individual level.
☐ True
☐ False
☐ I do not know

AW3: Healthcare clinical professionals work to prevent health problems from happening or recurring by implementing educational programs, recommending policies, offering preventive services, and conducting preventive research.

☐ True

☐ False

☐ I do not know

AW4: Public health professionals primarily treat individuals after they become sick or injured.

☐ True

☐ False

☐ I do not know

AW5: Public health works to limit health disparities by promoting healthcare equity, quality, and accessibility.

☐ True

☐ False

☐ I do not know

AW6: Public health functions may include:

		True	False	I do not know
AW6-1	Surveillance and monitoring of population health	☐	☐	☐
AW6-2	Health promotion and disease prevention	☐	☐	☐
AW6-3	Health protection for the population, including management of environmental, food, and occupational safety	☐	☐	☐
AW6-4	Preparedness and response to disease outbreaks, natural disasters, and other emergencies	☐	☐	☐
AW6-5	Assuring effective health governance, public health legislation, financing, and institutional support	☐	☐	☐
AW6-6	Training for specialists in curative medical aspects	☐	☐	☐
AW6-7	Assuring a sufficient and competent workforce for effective public health delivery	☐	☐	☐
AW6-8	Effective communication and social mobilization for health	☐	☐	☐
AW6-9	Advancing public health research to influence policy and practice	☐	☐	☐
AW6-10	Providing patients with acute treatment measures	☐	☐	☐

AW7: The new national healthcare model in Saudi Arabia primarily emphasizes public health aspects and practices.

☐ True

☐ False

☐ I do not know

PART THREE:
PERSONAL ATTITUDES TOWARD THE IMPORTANCE OF THE PUBLIC HEALTH MODEL

AT1: Some people think it is necessary to integrate the clinical healthcare model and the public health model to promote population health in Saudi Arabia, but others think the clinical healthcare model is sufficient. What is your opinion on this matter?

☐ The clinical healthcare model is sufficient to promote population health.

☐ Integration of both models is essential to promote population health.

AT2: Some people think that public health measures contribute significantly to preventing infectious diseases and many chronic diseases, but others think that public health measures have little impact. What is your opinion on this matter?

☐ Public health measures have little impact on preventing infectious and chronic diseases.

☐ Public health measures significantly help in preventing infectious and chronic diseases.

AT3: Some people think that public health measures have played a significant role in confronting the COVID-19 pandemic at the global and national levels, but others think their impact has been minimal. What is your opinion on this matter?

☐ Public health measures have played little or no role in confronting the COVID-19 pandemic.

☐ Public health measures have played a significant role in confronting the COVID-19 pandemic.

OPEN QUESTION:

Any additional information to enhance the results of the study?

Thank you for your participation in this study.

ملاحظة: يمكن استخدام هذا الاستبيان وتعديله دون إذن مسبق بشرط الإشارة للمصدر.

استبيان الوعي والمواقف تجاه نموذج الصحة العامة

استجابة لإصلاحات نظام الرعاية الصحية السعودي: دراسة مقطعية للوعي والمواقف تجاه نموذج الصحة العامة بين طلاب التخصصات الصحية

بسم الله الرحمن الرحيم

عزيزي طالب / طالبة التخصصات الصحية بجامعة جازان بالمملكة العربية السعودية
السلام عليكم ورحمة الله وبركاته، وبعد:
نحن مجموعة من الباحثين في جامعة جازان. هذا المسح يهدف لتقييم الوعي والمواقف بين طلاب التخصصات الصحية في جازان تجاه نموذج الصحة العامة كاستجابة للإصلاحات الحديثة للنظام الصحي السعودي، ونأمل منك التلطف بالمشاركة في هذه الدراسة.
نشكر لك اهتمامك، علمًا أن مشاركتك في هذه الدراسة هي مشاركة تطوعية ولا تتطلب أي معلومات شخصية، ومحتوى هذه الاستبانة سيستخدم لأغراض البحث العلمي فقط طبقاً لموافقة لجنة أخلاقيات البحث العلمي بجامعة جازا رقم REC42/1/145
إجابتك على كافة الأسئلة سيمكننا من التحليل الجيد لنتائج هذه الاستبانة. سيأخذ منك ملء الاستبانة حوالي 3 دقائق.

في حال الحاجة للاستفسار يمكنكم التواصل على البيانات التالية:
د. محمد بن جبران المالكي (الباحث الرئيس)
كلية الصحة العامة وطب المناطق الحارة
جامعة جازان
mjalmalki@jazanu.edu.sa

شكرًا لكم وبالله التوفيق.

1. هل أنت طالب في أحد التخصصات الصحية بجامعة جازان؟

☐ لا (لطفًا، بإمكانك التوقف هنا مع خالص الشكر)
☐ نعم (من فضلك انتقل للسؤال التالي)

2. أبلغ من العمر 18 عامًا أو أكثر، وأفهم هدف هذا البحث، وأوافق على المشاركة تطوعياً.

☐ لا (لطفًا، بإمكانك التوقف هنا مع خالص الشكر)
☐ نعم (من فضلك ابدأ الإجابة على أسئلة الاستبانة)

الجزء الأول: معلومات أساسية

أ. **النوع:**

☐ ذكر ☐ أنثى

ب. **العمر:** (__________)

ج. **كلية الدراسة:** (____________________)

د. **التخصص الدراسي:** (____________________)

ه. **المستوى الدراسي (السنة الدراسية):**

☐ السنة الدراسية الأولى
☐ من السنة الدراسية الثانية إلى ما قبل السنة الدراسية الأخيرة
☐ السنة الدراسية الأخيرة
☐ سنة تدريب الامتياز

و. **تلقيت تدريب في مجال الصحة العامة:**

☐ نعم ☐ لا

الجزء الثاني:
الوعي الشخصي تجاه نموذج الصحة العامة

(فضلًا الإجابة بحسب معرفتك دون الرجوع إلى أي مصادر علمية)

وعي1: الصحة العامة يمكن أن تعرّف بأنها علم حماية وتحسين صحة السكان بشكل عام من خلال تعزيز أنماط الحياة الصحية، وتحسين صحة البيئة، والوقاية من الأمراض المزمنة والمعدية والإصابات، والاستجابة للتهديدات الصحية، وإجراء البحوث الوقائية، والتوصية بسياسات الصحة العامة.

☐ صح
☐ خطأ
☐ لا أعلم

وعي2: الصحة العامة هي علم وممارسة تشخيص المرض وعلاجه والوقاية منه على المستوى الفردي.

☐ صح
☐ خطأ
☐ لا أعلم

وعي3: يعمل متخصصو الرعاية الصحية السريرية على منع حدوث المشكلات الصحية أو تكرارها من خلال تنفيذ البرامج التوعوية والتوصية بالسياسات الصحية العامة، وتقديم الخدمات الوقائية، وإجراء البحوث الوقائية.

☐ صح
☐ خطأ
☐ لا أعلم

وعي4: يركز متخصصو الصحة العامة بشكل أساسي على علاج الأفراد بعد أن يمرضوا أو يتعرضوا للإصابة.

☐ صح
☐ خطأ
☐ لا أعلم

وعي5: تعمل الصحة العامة على الحد من الفوارق الصحية من خلال تعزيز المساواة في الرعاية الصحية وجودتها وإمكانية الوصول إليها.

- ☐ صح
- ☐ خطأ
- ☐ لا أعلم

وعي6: من الوظائف الأساسية للصحة العامة:

		صح	خطأ	لا أعلم
وعي6-1	الترصد الوبائي والمراقبة الصحية	☐	☐	☐
وعي6-2	تعزيز الصحة والوقاية من الأمراض	☐	☐	☐
وعي6-3	ضمان الحماية الصحية للسكان من المخاطر البيئية والغذائية والمهنية	☐	☐	☐
وعي6-4	الجاهزية والاستجابة للطوارئ والأزمات والكوارث الصحية	☐	☐	☐
وعي6-5	ضمان الحوكمة الصحية الفعالة وتشريعات الصحة العامة والتمويل والدعم المؤسسي	☐	☐	☐
وعي6-6	تدريب المتخصصين في المجالات الطبية العلاجية	☐	☐	☐
وعي6-7	ضمان قوة عمل كافية ومتنوعة ومؤهلة لتقديم خدمات الصحة العامة	☐	☐	☐
وعي6-8	التواصل الفعال لإعلام وتثقيف الناس والتعبئة الاجتماعية من أجل الصحة	☐	☐	☐
وعي6-9	النهوض بأبحاث الصحة العامة للتأثير على السياسات والممارسات	☐	☐	☐
وعي6-10	تقديم الإجراءات العلاجية العاجلة للمرضى	☐	☐	☐

وعي7: يركز نموذج الرعاية الصحية الوطني الجديد في المملكة العربية السعودية بشكل أساسي على جوانب وممارسات الصحة العامة.

- ☐ صح
- ☐ خطأ
- ☐ لا أعلم

الجزء الثالث:
المواقف الشخصية تجاه أهمية نموذج الصحة العامة

مواقف1: يعتقد البعض أنه من الضروري تكامل نموذج الرعاية الصحية السريرية ونموذج الصحة العامة لتعزيز صحة السكان في المملكة العربية السعودية، لكن يعتقد البعض الآخر بكفاية نموذج الرعاية الصحية السريرية. ما رأيك في هذا الامر؟

- ☐ يكفي نموذج الرعاية الصحية السريرية لتعزيز صحة السكان
- ☐ تكامل كلا النموذجين مهم لتعزيز صحة السكان

مواقف2: يعتقد البعض أن تدابير الصحة العامة تساهم بشكل كبير في الوقاية من الأمراض المعدية والعديد من الأمراض المزمنة، لكن يعتقد البعض الآخر أن تدابير الصحة العامة ذات تأثير ضئيل على هذه الجوانب. ما رأيك في هذا الامر؟

- ☐ تدابير الصحة العامة لها تأثير ضئيل على الوقاية من الأمراض المعدية والأمراض المزمنة
- ☐ تساعد تدابير الصحة العامة بشكل كبير في الوقاية من الأمراض المعدية والأمراض المزمنة

مواقف3: يعتقد البعض أن تدابير الصحة العامة تلعب دورًا رئيسيًا في مواجهة جائحة كوفيد-19 على المستويين العالمي والوطني، لكن يعتقد البعض الآخر أن تأثيرها يعد معدوماً أو ضئيلاً. ما رأيك في هذا الامر؟

☐ تلعب تدابير الصحة العامة دورًا ضئيلًا أو معدومًا في مواجهة جائحة كوفيد-19

☐ تلعب تدابير الصحة العامة دورًا رئيسيًا في مواجهة جائحة كوفيد-19

4: أي معلومات إضافية لتعزيز نتائج الدراسة؟

شكراً لكم على المشاركة في هذه الدراسة
